# A Data-Driven Simulator for the Strategic Positioning of Aerial Ambulance Drones Reaching Out-of-Hospital Cardiac Arrests: A Genetic Algorithmic Approach

**DOI:** 10.1109/JTEHM.2020.2987008

**Published:** 2020-04-21

**Authors:** Conor Mackle, Raymond Bond, Hannah Torney, Ronan Mcbride, James Mclaughlin, Dewar Finlay, Pardis Biglarbeigi, Rob Brisk, Adam Harvey, David Mceneaney

**Affiliations:** 1 School of ComputingUlster University 2596 NewtownabbeyBT37 0QBU.K.; 2 HeartSine Technologies Ltd.BelfastBT3 9EDU.K.; 3 Southern Health and Social Care TrusPortadownBT63 5QQU.K.; 4 School of EngineeringUlster University 2596 NewtownabbeyBT37 0QBU.K.; 5 Department of CardiologyCraigavon Area Hospital 155304 PortadownBT63 5QQU.K.

**Keywords:** OHCA, AED, UAV, ambulance drone

## Abstract

Objective: The Internet of Things provide solutions for many societal challenges including the use of unmanned aerial vehicles to assist in emergency situations that are out of immediate reach for traditional emergency services. Out of hospital cardiac arrest (OHCA) can result in death with less than 50% of victims receiving the necessary emergency care on time. The aim of this study is to link real world heterogenous datasets to build a system to determine the difference in emergency response times when having aerial ambulance drones available compared to response times when depending solely on traditional ambulance services and lay rescuers who would use nearby publicly accessible defibrillators to treat OHCA victims. Method: The system uses the geolocations of public accessible defibrillators and ambulance services along with the times when people are likely to have a cardiac arrest to calculate response times. For comparison, a Genetic Algorithm has been developed to determine the strategic number and positions of drone bases to optimize OHCA emergency response times. Conclusion: Implementation of a nationwide aerial drone network may see significant improvements in overall emergency response times for OHCA incidents. However, the expense of implementation must be considered.

## Graphical abstract

The paper hypothesises that a network of drone AEDs can significantly reach more people having an out of hospital cardiac arrest in a shorter amount of time. This would increase the chances of survival and lead to better outcomes. The graphical abstract illustrates our development of a drone network where we optimise the geolocation of drone stations using a genetic algorithm. We compare the response times of these drone AEDs with the response times of nearby ambulances and lay rescuers who might access nearby public accessible AEDs.

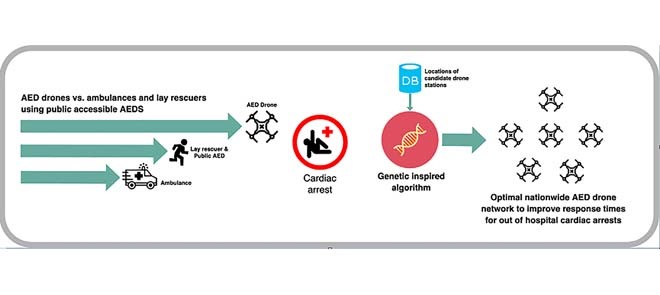

## I. Introduction

The Internet of Things (IoT) is the connection of devices across a network, where each device is uniquely identified and can send and receive data. These devices consist of sensors, actuators, internet connectivity and artificial intelligence (AI). The market for IoT will go from 212 billion USD in 2019 up to 1.6 trillion USD by 2025 [1]. Smart cities use IoT technology to enhance their efficiency, sustainability, economic development and the quality of life for their citizens. There are a number of examples for smart cities worldwide, with Hong Kong, Toronto, New York, Stockholm and London being amongst the top smart cities in the world [2]. These smart cities can benefit from improvements in energy efficiency, infrastructure, transportation, public safety, water treatment, healthcare and a number of other areas.

Healthcare will see an improvement for patient care due to the ever-increasing amount of patient data and personal smart devices. Ambulance drones have been prototyped to carry an Automated External Defibrillator (AED) to target the major problem of reaching people who suffer from Out-of-Hospital-Cardiac-Arrests (OHCA) as fast as possible. Early intervention is necessary as there is a strong association between early defibrillation and increased survival [3]. It is hypothesized that, in some cases, these drones can reach OHCA patients faster than an ambulance or even a bystander utilizing a publicly accessible AED [4].

There are approximately 30,000 cases of suspected cardiac arrests that receive a resuscitation attempt by the NHS annually in the United Kingdom [5]. The majority (60.8%) of OHCAs occur at home with only 2.3% of patients being treated with a publicly accessible AED prior to the arrival of emergency medical services [6]. A cardiac arrest occurs when the heart stops beating unexpectedly. It may be triggered by electrical malfunctions in the heart. As a result, there will be an insufficient supply of oxygenated blood to the brain, lungs and vital organs, hence the patient will rapidly lose consciousness. Death will occur within minutes if the patient does not receive effective cardiopulmonary resuscitation (CPR) and rapid defibrillation if necessary [7]. A heart attack may also trigger a cardiac arrest [7]. A heart attack, or acute myocardial infarction, is when the blood flow to the heart is blocked causing ischaemia. Heart attack symptoms may occur for minutes, hours or days. Therefore, a patient can have a limited amount of time to contact the emergency services. Chances of survival following cardiac arrest decrease with time. Patients can have up to 50-70% chance of survival if they receive defibrillation within the first 3–5 minutes [8]. The chance of survival then decreases significantly with every minute that passes after 5 minutes [9]. The current protocol for treating a patient in cardiac arrest is immediate CPR and rapid defibrillation by a paramedic or a bystander using a nearby public AED. Publicly accessible AEDs are typically situated in places with high footfall such as supermarkets, tourist areas, schools, offices and sports centers.

In Northern Ireland, Category A emergency calls are defined as life-threatening and should be responded to within 8 minutes. This category includes cardiac arrests. However, in 2018 only 41.9% of Category A calls were responded to within the 8-minute target [10]. This is a worrying statistic considering OHCA patients rely on rapid response times to increase their chances of survival. However, first responder programmes such as the Good Sam mobile app [11] provide the locations to access nearby publicly accessible AEDs as well as alerting nearby trained responders. The Good Sam app is being used in Northern Ireland through a collaboration with the Northern Ireland Ambulance Service (NIAS) with the aim to improve OHCA response times. Ambulance drones may offer a further improvement in combination with traditional emergency medical services (EMS) and first responder programmes (Good Sam app). Ambulance drones which can carry an AED have been prototyped by Delft University of Technology in the Netherlands. These drones cost around \\(15,000 and can travel up to 100 km/h (62mph). They weigh 4 kg with a built-in defibrillator and can carry an additional payload of 4kg. The drone can fly autonomously when given GPS coordinates and has a built-in live camera and speaker to allow an emergency operator to communicate with the caller in real-time to provide CPR instructions or other medical advice [12].

The aim of this study is to experiment with a use-case of drone-delivered AEDs using a computer simulation program that is driven using real world data. The computer program will determine the optimum drone base locations and coverage for drone delivered AEDs across a country (Northern Ireland) to improve response times for OHCA incidents. The program will compare the drone response times with the response times from ambulance services and response times from a bystander accessing a nearby publicly accessible AED.

The simulator uses several heterogenous datasets and a Genetic Algorithm to determine the strategic number and positioning of drone bases to optimize emergency response times. The study investigated the following research question:

• To what extent can drone-delivered AEDs reduce OHCA emergency response times according to a simulator that is driven using real world data?

## II. Research in the Area

We investigated three drone options which are potentially suitable for the delivery of AEDs (*Table 1*). The ambulance drone prototype developed by the *Delft University of Technology* is specifically designed for the transportation of a built-in AED to OHCA patients. There are other drone prototypes being researched that would be capable of transporting an AED. The Defikopter developed by a German non-profit group *Definetz* offers the transportation of an external AED which is delivered to a patient using GPS coordinates. This drone can reportedly travel at 70km/h (43mph) and costs reportedly around $26,000 [13]. Amazon have also developed a fully autonomous drone which can fly up to 15 miles with a given payload of under 2.2kg [14].

**TABLE 1:** Drone Specifications

Manufacturer	Cost	Available AED	Max Speed	Payload	Range
Tu Delft	£15,000	Yes	100 km/h	4kg	12 km
Definetz	$26,000	Yes	70 km/h	–	10 km
Amazon	–	No	80 km/h	2.2kg	24 km

The combination of these lightweight AEDs with current aerial drone technology demonstrates the potential for future manufacturers. The lightweight HeartSine AED combined with the Amazon drone would enable the delivery of an AED to an OHCA patient within a 24km radius at 80km/h offering further delivery of an AED than the Tu Delft ambulance drone. Given the research undertaken in drone deliveries, it is apparent that the next generation of drones may allow for the prompt and safe delivery of AEDs to OHCA patients.

Fleck *et al.* conducted a case-study to understand the ability of manufacturers to include a defibrillator on a lightweight drone whilst maintaining quick and efficient treatment for an OHCA patient. The authors conducted a comparison of Schiller FRED EasyPort (600 grams) and the HeartSine Samaritan PAD 300P (1100 grams) AEDs. The study carried out OHCA simulations to determine the usability of the AEDs for untrained users. Although the Schiller AED was more light-weight, it was deemed more complicated and less user-friendly than the HeartSine AED. It was concluded that clear verbal instructions were important to ensure correct defibrillation and reduced time-to-shock delays [15].

Lennartsson has conducted research into the strategic positioning of ambulance drones delivering defibrillators to OHCA patients in Stockholm County, Sweden [4]. Each of the drone bases had a radius of 10km and were decided based on where the most OHCAs occurred. The Stockholm county area is 6522 km^2^ and 10 bases were selected. An overview of the results is described in *Table 2* indicating the extent to which response times were reduced. Within an 8.5-minute radius there were reductions of up to 22 minutes, in one case reducing from 31 to 9 minutes, with an average reduction of 6.15 minutes. In a 5-minute radius there were reductions up to 17 minutes, e.g. reducing from 22 minutes down to 5 minutes with an average reduction of 5.4 minutes. The drone locations within a 3-minute radius had reductions up to 19 minutes, reducing from 32 to 3 minutes and an average of an 8-minute reduction. This provides an indication that the drone networks have the potential to be successful in reducing response times.

**TABLE 2:** Stockholm OHCA Response Improvements Using Ambulance Drones

Radius	Smallest Improvement	Largest Improvement	Average Improvement
8.5 min	−1 minutes	22 minutes	8 minutes
5 min	3 minutes	17 minutes	5.4 minutes
3 min	4 minutes	29 minutes	8 minutes

This research has involved the development of a real world data-driven simulator to estimate the potential improvement in OHCA response times by implementing an AED drone network.

## III. Methodology

This study involved hosting an Ubuntu Linux server using Amazon’s Elastic Compute Cloud (EC2) on Amazon Web Services and building a simulator using the Python programming language. The simulator uses a number of datasets: 1) an open dataset from the Northern Ireland Ambulance Service which specifies the geolocation of publicly accessible AEDs in Northern Ireland [16]; 2) open datasets from Northern Ireland Council and Voluntary Action (NICVA) which specifies the geolocation of local ambulance stations and General Practice (GP) clinics [17], [18]; 3) a synthetic dataset generated by the author of probabilistic geolocations of 10,000 OHCA incidents; 4) two datasets from Northern Ireland Statistics and Research Agency (NISRA) which specify the geolocation of police and fire emergency stations that can be potential locations for operating AED drones. Given the law of diminishing returns we can determine the point at which the response times from adding additional drones will begin to plateau and therefore become less cost effective. This enables us to select a subset of dataset 4 which is optimized using a genetic algorithm to determine an optimal number of drones for reducing response times.

### A. Drone Specifications

For this research we used the *Delft University of Technology’s* drone specifications due to its specific design for AED transportation. This drone is capable of travelling within a 12km radius on a single battery charge with an additional payload of 4kg. It has a built-in camera and can travel at 100km/h, and is fully autonomous which will be assumed throughout this study [19].

### B. OHCA Incidents

To help simulate the most accurate distribution of incidents for the OHCAs, we used the distribution of the days and time-of-days of real-world OHCA incidents. This distribution was provided by HeartSine, a manufacturer of public accessible AEDs. The electronic AED event data was downloaded by the end user and submitted to HeartSine for inclusion in their post market clinical follow-up data collection [20].

**FIGURE 1.**
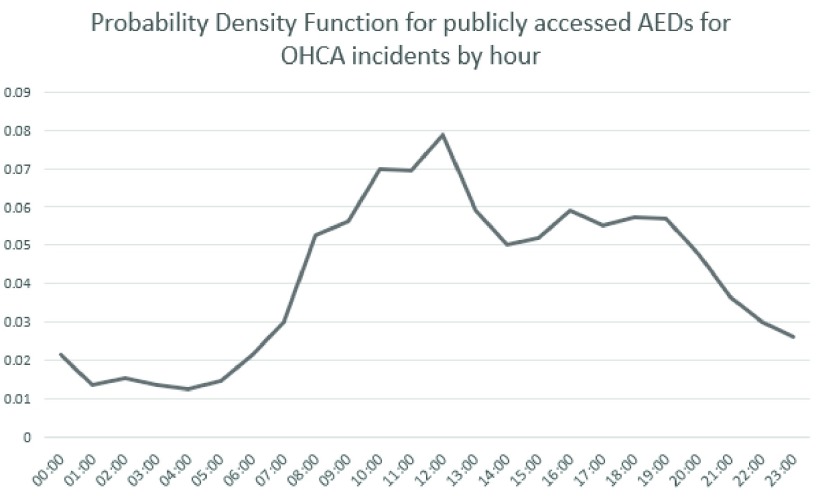
Probability density function for publicly accessed AEDs for OHCA incidents by hour.

**FIGURE 2.**
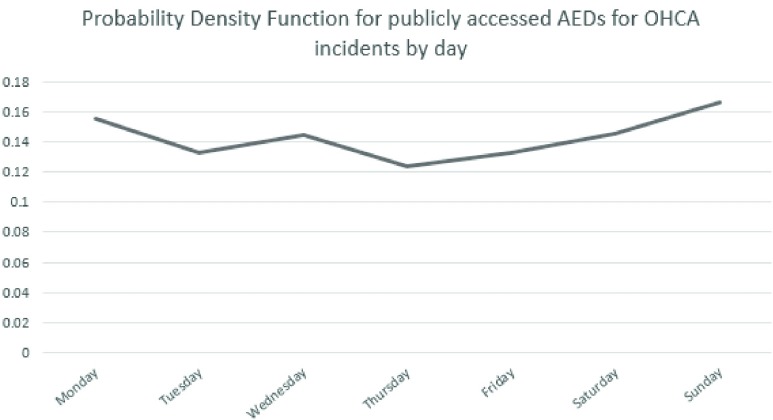
Probability density function for publicly accessed AEDs for OHCA incidents by day.

The probability density functions are shown in *Figure 1* and *Figure 2* and are used in the simulator to help distribute the OHCA incident simulations in this study.

### C. Northern Ireland Health and Social Care Trusts

**FIGURE 3.**
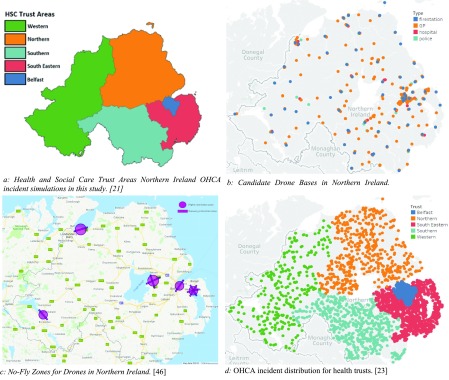
Map plots for Northern Ireland.

We estimated the number of drone bases for each Health and Social Trust in Northern Ireland (HSCNI). There are five trusts, as shown in *Figure 3a*: Northern, Western, South Eastern, Southern and Belfast trusts. *Figure 3a* was created using a Northern Ireland trust boundaries shape file accessed from OpenDataNI [21] and the population density for each trust is publicly available from Northern Ireland Statistics and Research Agency [22] and is reported in Table 3. The number of simulated OHCAs have been distributed between the trusts based on their population density.

**TABLE 3:** Distribution of Simulations Based on Population Density in Northern Ireland

HSCT	Population Density in Northern Ireland	No. of Simulations
Belfast	77.30%	7730
Northern	4.91%	491
South Eastern	9.82%	982
Southern	5.24%	524
Western	2.73%	273

### D. Candidate Drone Bases

For this study, all police stations, fire stations, hospitals and general practice (GP) clinics in Northern Ireland were included as candidate AED drone bases. These locations were selected since they are in secure locations comprising of professionals capable of maintaining and operating drones (at least this is the assumption made in this study). This included a total of 242 potential locations for the AED drone bases shown in *Figure 3b* which is demonstrated using Tableau mapping tools [23].

### E. No-Fly Zones

There are five drone flight restriction areas which make it illegal to fly a drone within a 5km radius or along the takeoff or landing zones unless the user of the drone has the required permissions [24]. These restricted zones as shown in *Figure 3c* were taken into consideration for the data-driven simulator when calculating response times after the ambulance drone bases are included.

### F. Generating Geolocation Points

There are no publicly available OHCA incidents for Northern Ireland. Therefore, demonstrative data was generated using randomly selected coordinates to represent OHCA incidents in each trust. The distribution of 10,000 generated points is shown in *Figure 3d* using Tableau mapping tools [23] and in *Table 3* across all health trusts, and were determined through the use of the Geocoding API from the Google Maps Platform [25]. These selected points are only in residential roads or addresses, and excluding places such as within lakes, mountains and other extreme locations.

**FIGURE 4.**
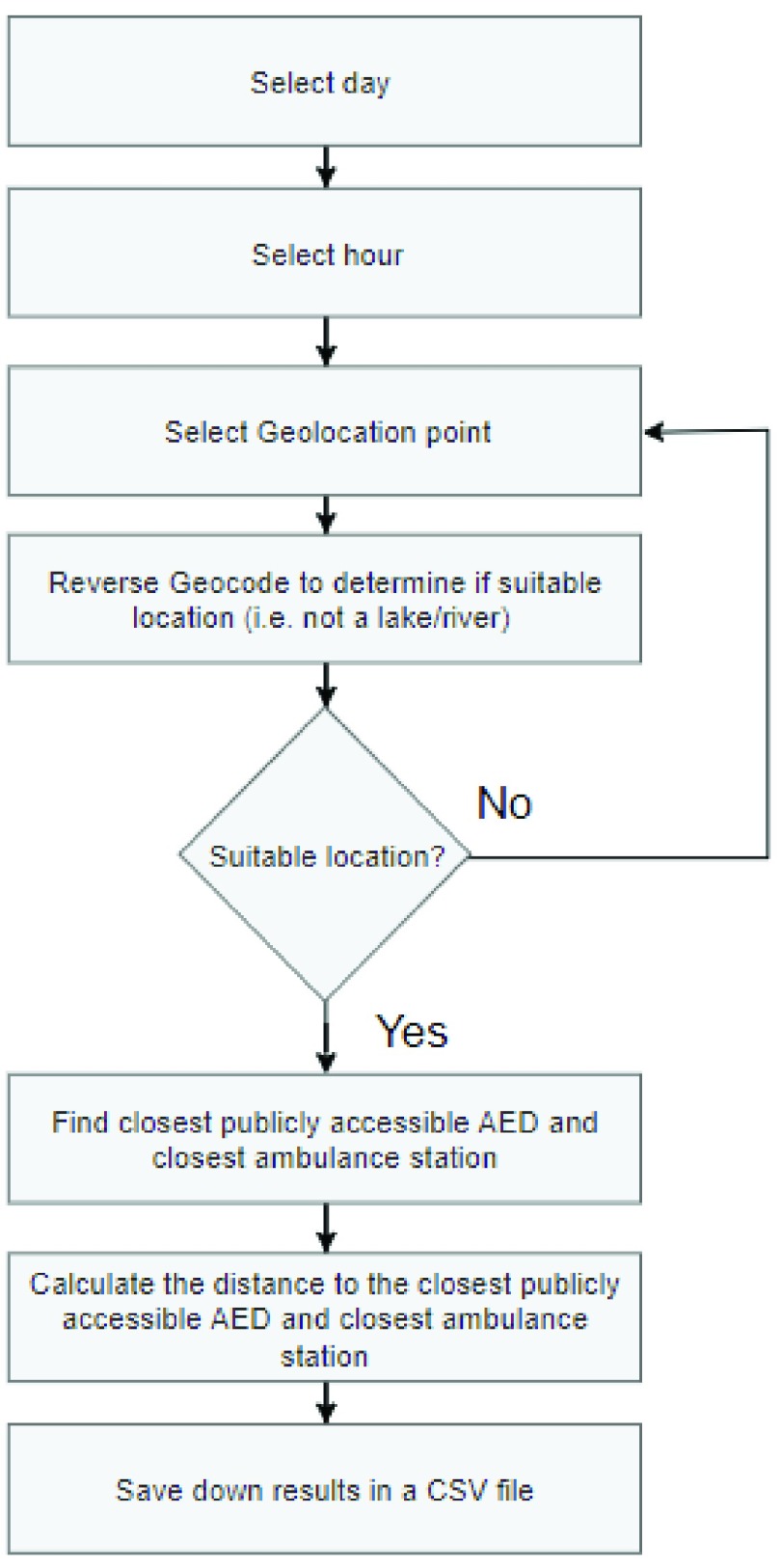
Generating points flow chart.

The Haversine formula was used to find the distance from the generated OHCA location and the nearest ambulance station and the nearest publicly accessible AED. A day and hour was generated for each OHCA location using the probability functions provided by HeartSine. These generated locations are saved as a CSV file to ensure consistent results throughout the research. The flow chart for generating geolocations is shown in *Figure 4.*

Haversine formula (equation 1):

\begin{align*} d=2r\arcsin \left({\surd \left({\frac {\varphi _{2}-\varphi _{1}}{2}}\right)+\cos (\varphi _{1})\cos (\varphi _{2})sin^{2}\left({\frac {\lambda _{2}-\lambda _{1}}{2}}\right)}\right) \!\!\!\! \\\tag{1}\end{align*}

*where* $d$ is the distance between two points on a sphere, $r$ is the radius of the sphere (earth’s radius = 6356km), $\varphi _{1}$ and $\varphi _{2}$ are the origin and destination latitude points in radians and $\lambda _{1}$ and $\lambda _{1}$ are origin and destination longitude points in radians we can determine the distance between two points on earth given their longitude and latitude [26].

### G. Simulations

These probabilistic OHCA geolocations and times were used to compute travel response times for: 1) a nearby ambulance to arrive at the scene, 2) a bystander to obtain a publicly accessible AED, and 3) for the ambulance drone to arrive at the scene (once the drone bases have been strategically selected using the genetic algorithmic approach). The Google Maps Platform with the Distance Matrix API [25] were also used to account for vehicular traffic. To represent a response time from a blue light ambulance dispatch, the time travelled was reduced by 25%. Simulating the journey to reach a publicly accessible AED, the Distance Matrix API was used again but for driving to and from the public AED location. To simulate the drone journey, the Haversine formula was used.

### H. Genetic Algorithm for Positioning Drones

The Genetic Algorithm (GA) is a heuristic stochastic search algorithm inspired by Darwin’s theory of natural selection. GAs run through a specified number of iterations (generations) whilst retaining the fittest solutions on each iteration. The aim is to repeat this process until an optimal solution is achieved. GAs consist of six main components; a gene (i.e. a drone base), a chromosome (i.e. a set of drone bases), a population (multiple chromosomes), crossover, mutation and a fitness test. The population is a collection of chromosomes and each chromosome is a collection of genes. Each generation for the GA is the population inclusive of the two fittest chromosomes from the previous generation. The crossover operation is applied at each generation by joining pairs of chromosomes. Mutation ensures genetic diversity from one generation to the next by considering a set probability of occurrence. GAs have been widely used in different search and optimization problems due to their affordable computational costs and ability to overcome non-linear characteristics of a problem while looking for global optimal solutions [27]. In studies on developing new technologies towards smart cities using IoT, GAs have been used diversely. Examples include: improving the energy efficiency of indoor buildings [28], algorithms for parking space locations [29], optimizing logistics and procedures and route identification for waste collection [30], in decision support systems for managing road networks during natural disasters [31], designing optimal sewer networks [32], optimizing and managing risk and routing of contaminated healthcare textiles [33].

In this study, GAs are used to strategically position drone bases to optimize the OHCA response times. The initial population of GAs are generated with random drone bases (genes) to make up a set number of chromosomes (a drone base network). After the initial population is generated, the two fittest chromosomes are retained and then ‘crossed over’ to create a new chromosome. This same crossover process occurs with the rest of the pairs of chromosomes in descending order of fitness, with mutation occurring 10% of the time after a crossover to ensure genetic diversity. Additional randomly generated chromosomes are added within each generation to ensure the population size remains the same each time, and then the individual chromosome fitness is calculated. This process will happen for a set number of generations to leave the remaining fittest chromosome with the optimum drone base network from all generated chromosomes based on the fitness of travel times to OHCA incident locations.

### I. Fitness Test

**FIGURE 5.**
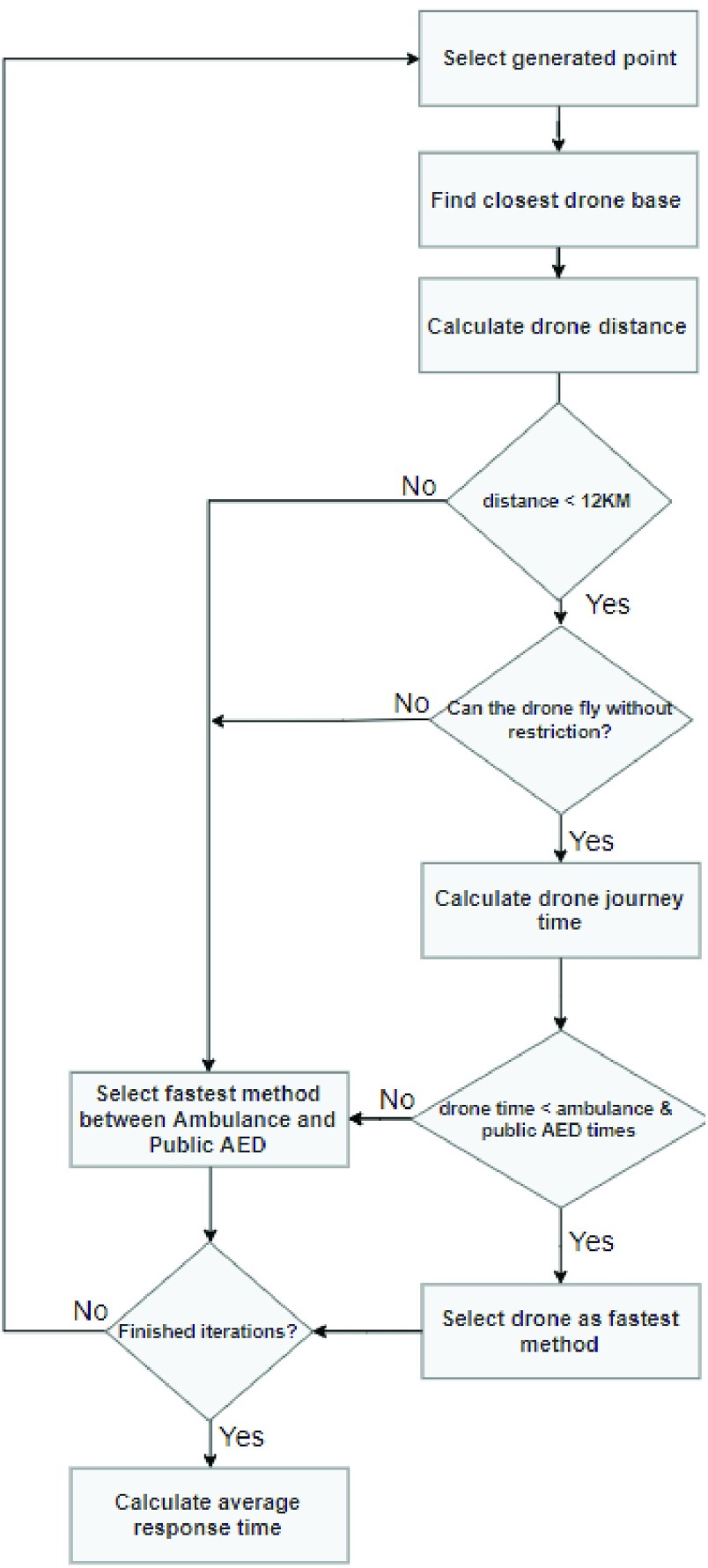
Fitness test simulations.

To determine the fitness of each chromosome, the average response time for the emergency services was calculated with the selected drone bases included to reach the simulated OHCA incidents. *Figure 5* shows the flow chart that illustrated the algorithm iterating through generated OHCA locations and finding the fastest method of emergency response. Given that the drone cannot travel further than 12km, in the case where the closest drone base is over that limit, this will be excluded and the next fastest method will be accepted. Once all iterations for OHCA incidents are complete, the average response time is calculated, and this is then the fitness indicator for the chromosome (i.e. set of geolocated drones).

## IV. Results

The results of this study demonstrate the difference in emergency response times for OHCA incidents throughout Northern Ireland’s health and social care trusts. The results depict the response times before a drone network is implemented including only ambulances and public AEDs, and after a drone network is implemented including ambulances, public AEDs and ambulance drone responding to incidents.

### A. Final Drone Bases (N = 78) Selected for Northern Ireland

**FIGURE 6.**
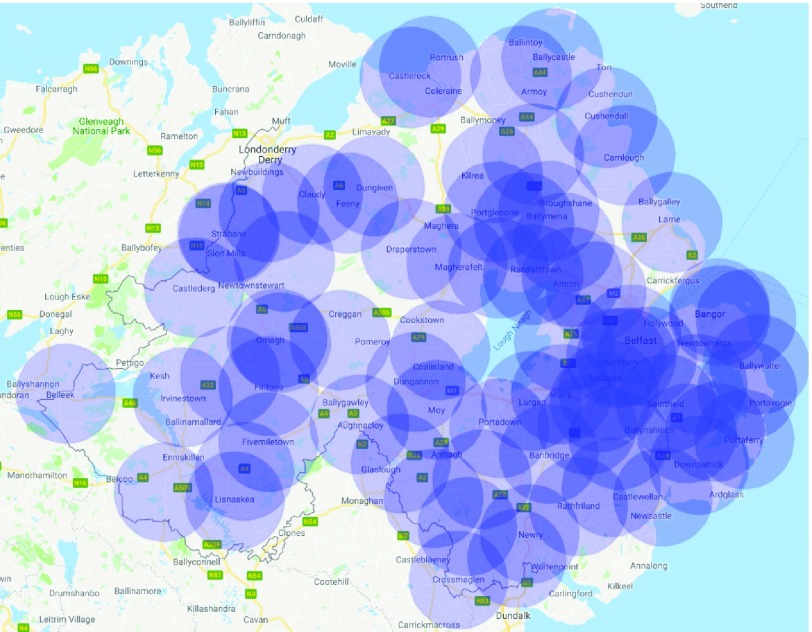
Final drone base locations in Northern Ireland.

**FIGURE 7.**
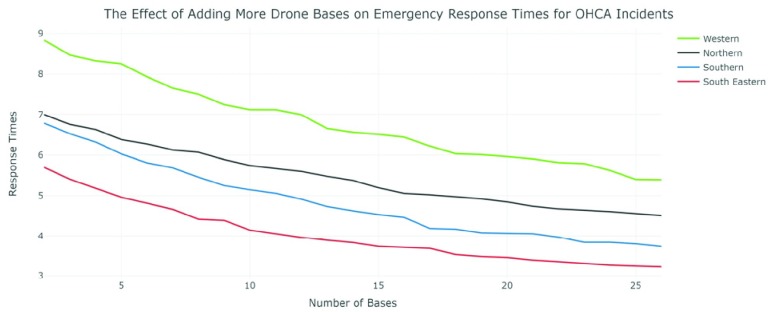
The effect of adding more drone bases on emergency response times for OHCA incidents.

*Figure 6* is the final distribution of drone bases across Northern Ireland as determined using the Genetic Algorithm. *Figure 7* shows the effect of adding more drone bases to the average response times for each trust except Belfast as it was already heavily saturated with OHCA response options and seen limited improvements using any more than 3 drone bases. The chosen model includes a total of 78 drone bases.

### B. Time Group Reductions After Drone Network Implemented

**FIGURE 8.**
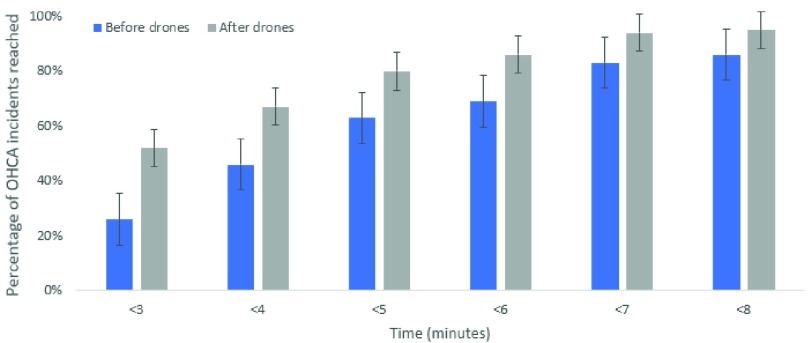
OHCA response time groups after drone network implemented.

*Figure 8* describes the total percentage of OHCA incidents reached at each time interval from less than 3 minutes up to less than 8 minutes. Whilst there is an improvement at each interval, the greatest improvement is for incidents that can be reached in under 3 minutes. Specifically, there are over double the number of incidents reached in under 3 minutes after the simulated drone network is implemented.

### C. Response Time Improvements Across Each Health and Social Care Trust

**FIGURE 9.**
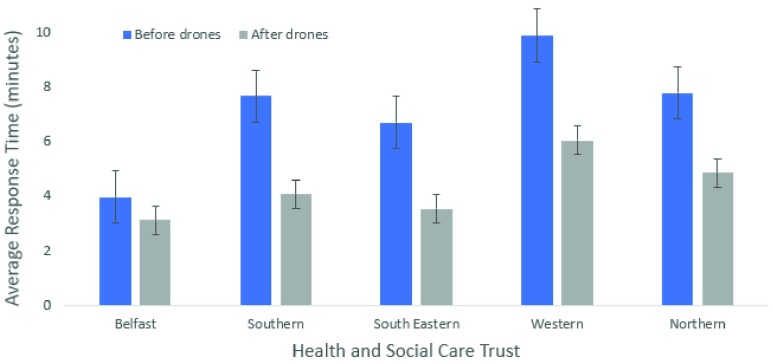
Comparison of average response times before and after ambulance drone network for health and social care trusts.

*Figure 9* shows the improvement in the average response times for each social care trust area (p<0.05). The average time is only slightly reduced below the 3-minute mark in Belfast, but this is due to a larger population density in a smaller area with a greater number of emergency resources already available. The other trusts have more rural areas with a greater distance from an ambulance station or publicly accessible AED. These trusts have seen around a 50% improvement in response times after implementing a drone network. This may significantly increase the chance of survival for people living in more rural areas.

### D. Emergency Service Count for OHCA Incidents Before and After Simulated Drone Network Implemented

**FIGURE 10.**
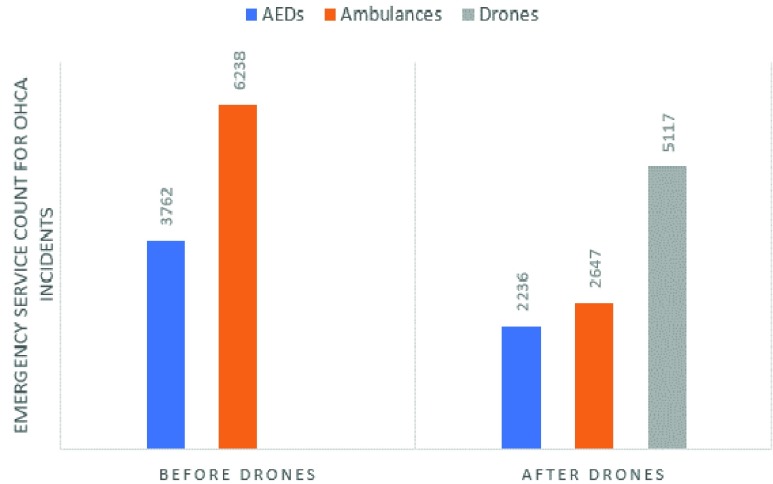
Emergency service count for OHCA incidents before and after drone network is implemented.

*Figure 10* displays the total count for each type of emergency service (publicly accessible AED, ambulance and drone) before and after a drone network is implemented. After the drone network was implemented publicly accessible AEDs made up 19.74% of responses, ambulances made up 25.66% and drones made up 54.6% of total responses.

### E. Average Response Times Boxplot Graph Before Drone Network Implemented

**FIGURE 11.**
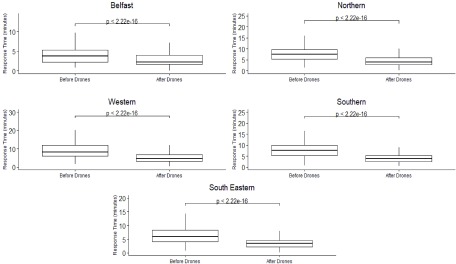
Boxplots for health and social care trusts response times before and after simulated ambulance drone network is introduced (differences in the before and after response times were assessed using a Wilcoxon test).

*Figure 11* shows the boxplots of response times (minimum, first quartile, median, third quartile and the maximum) for each social care trust before and after the ambulance drone network has been implemented. These boxplot results will change accordingly to the number of drone bases selected in the ambulance drone network. The Wilcoxon test highlights that there is a significant difference in the response times before and after the drone network is included with the median being significantly reduced in each trust area (p<0.001).

## V. Discussion and Conclusion

This paper used the unique approach of using a data-driven simulator with a Genetic Algorithm to optimally select drone base geolocations throughout an entire country and a data-driven simulator to estimate the potential reduction in response times for OHCA incidents. The results show significant improvements in response times for OHCA incidents when implementing a drone network. The drones also offer a dedicated response to these incidents and a shorter response time for rural incidents which is likely to improve the chances of survival for many cases. To implement a drone network throughout nationwide with similar results would be very costly. However, the cost of life outweighs the cost of implementing the network. The drone network described in this paper is an example of an efficient solution, but this could be altered dependent on budget and success of trials with a smaller number of drones. The data-driven simulator is useful for estimating the potential benefit of implementing the drone network, but its accuracy is limited without trial emergency drone flights.

### A. Ambulance and Public AED Resource Consideration

Further to consider are the limitations of ambulance resources across emergency incidents. According to the Northern Ireland Statistics Research Agency, there is a response time of 12+ minutes across the whole of Northern Ireland [22]. It is also important to note that according to the simulations carried out, some rural residential areas could take 25+ minutes to receive an ambulance from the closest ambulance service station. Category A Response calls are not always OHCA incidents, but ambulance drones are specifically designed for OHCA incidents. This ensures a high chance of availability for an OHCA patient to receive rapid response for a drone even at busy times.

The simulator results show publicly accessible AEDs being used just over 37% of the time in OHCA cases before drones were introduced and just over 19% after the drone network was implemented. However, the Out-Of-Hospital Cardiac Arrest Outcomes (OHCAO) Registry claim publicly accessible AEDs are used only 2.4% of the time [6]. This is likely caused by distance from the nearest AED, the lack of bystanders nearby available to assist, lack of transport option available to reach the nearest publicly accessible AED or being unaware of the nearest one.

### B. Cost of the Drone Network

With the lifespan of a drone being four years and each drone costing \\(15,000, the total cost to implement the drone network including 20% of the purchase price for annual maintenance, is approximately $2.11million [12], [34]. In a cost-effective analysis of utilizing AED drones in North Carolina, US, Bogle *et al.* [34] concluded that in a large scale network, AED drones have the potential to improve OHCA survival rate while being cost-effective. However, due to the wide range of assumptions taken in that study, it is important to undertake a detailed cost-effective analysis for AED drone networks considering personnel training and maintenance.

### C. Limitations

Drones are currently a developing technology with many restrictions limiting their commercial capabilities. One major restriction is their battery capacity limiting their travel times and reducing their available uptime. This is likely to improve significantly in the next few years of development with upcoming wireless charging hotspot technology currently being researched and prototyped [35]. An additional limitation is the flight restrictions in which drones must not be flown. Northern Ireland have 5 registered no-fly zones around airports. There are also strict rules on drones always remaining within line of sight for the user and at least 50 meters from buildings, vehicles or large crowds and never above private residential areas. These regulations will slow down the progress of implementing an emergency services autonomous drone network. However, as this is a relatively new technology, with increased research and development it is hoped that necessary licenses will be introduced to tackle these problems for emergency unmanned aerial vehicles (UAV). Public trust in new technologies is a concern as there may be an initial discontent in the use of emergency UAVs. However, perhaps the tradeoff between public trust and the ability for emergency UAVs to save lives will be too great to not consider implementation. The weather is another factor to consider for drone capabilities. It may be difficult to successfully implement a drone network with the unpredictable weather conditions present in Northern Ireland. The ambulance drone used would require weather resistant technology to ensure the successful delivery of an AED to an OHCA incident.

The simulations carried out for this research are based on ideal conditions taking into consideration factors such as traffic. The model assumes that there are no co-occurring OHCA incidents in the same region that require the same drone. In the last year in Northern Ireland there have been over 1,400 cardiac arrests that occurred in the community outside a hospital environment [36]. It has been assumed with 78 drone bases that the probability of two incidents happening at the same time in the same area is low. However, the GA model is limited as concurrent OHCA incidents are not considered. Consequently, there may be differences in actual response times dependent on factors such as emergency service resources, a delayed drone response in an area caused by concurrent OHCA incidents, or the recharging of drones between incidents. The simulations also excluded areas including mountains and lakes due to the limitations of the Google Maps API for calculating journeys where there are no roads.

Furthermore, the model assumes drone reliability and did not consider a safe landing area for the AED drone. Ideally, ambulance drones should land less than 5 minutes walking distance away from the OHCA patient. Also, even small drones need up to 150 m of space, and preferably flat ground for a safe landing [37]. Accordingly, acquiring an embedded vision system for landing site detection can be one solution to address this obstacle. These systems normally consist of a vision marker and sensor along with an embedded system which holds a deep learning algorithm, such as a Convolutional Neural Network to detect and position the marker [38], [39].

### D. Ethical and Societal Challenges

Different studies have experimented using mock AED drones in rural [40] and community [41], [42] settings to assess participant confidence in extracting the AED from the drone and using it with guidance. In all cases, most of the participants felt confident and had low or no safety concerns. This suggests the feasibility of using the emergency UAVs. However, there are several social and ethical challenges. Safety and security are a concern for the technology used, the user in control and the public. *Montgomery et al*. discuss the vulnerabilities of drones being captured and controlled vis GPS spoofing or signal jamming [43]. These vulnerabilities can result in a drone losing control and crashing, risking public safety and damage to the drone. Most importantly, the drone is at risk of not reaching its destination which will significantly impact the patient’s survival outcomes. Ambulance drones would almost certainly require flying over public and private areas with a built-in camera capturing video data for the entirety of its flight and once it is with the OHCA patient. This will result in highly sensitive information being vulnerable to hackers intercepting the data mid-flight or from the drone base. *Tarun Rana et al.* describe stronger encryption methods using Blockchain technology with Private and Public key cryptography [44]. These methods or similar will be necessary to provide optimal protection to defend against these types of security risks. Noise pollution is another issue to be considered, although ambulance drones will not be as common an occurrence as delivery drones would be. There is also the impact of health and social care trusts declining to implement a network of emergency drones which could result in loss of life from fatal OHCA incidents that are potentially avoidable if ambulance drones are available. However, there will be a trade-off between budget and availability. Extending the drone network will be expensive until drone technology become widely produced and benefits from economies of scale are demonstrated. Due to the high value of equipment necessary for an ambulance drone with an included defibrillator, theft is a major concern and must be considered. It would be necessary to take preventative measures to avoid the theft of ambulance drone equipment.

Overall, benefiting from UAVs in OHCA occurrences, apart from technology, requires community or governmental ownership and consequently laws and regulations, training protocols, licensing, insurance and social awareness [45]. A study with focus on stakeholder analysis can highlight the concerns behind using this technology.

### E. Further Research

Future research could consider the effect of drone AED delivery on survival outcomes. Due to the considerable expense of implementing a drone network in Northern Ireland, this would be necessary prior to implementation. It would be also beneficial to include extreme areas, such as lakes and mountains, in the simulations as OHCA incidents will also occur in these places, and drones may be a better alternative for these situations than the current strategies being utilized. It would also be beneficial to gather OHCA data from all AED manufacturers rather than only HeartSine’s AED data. Furthermore, when finding an optimal drone network, it would be necessary to obtain the locations and times of real OHCA cases in Northern Ireland which are not currently publicly available. This would help indicate high risk areas and provide more accurate information on response times from ambulances and publicly accessible AEDs to assist the simulator in finding the most strategic positioning for the ambulance drone network.

It is anticipated that drone technology will improve in the future and it is recommended to undertake further research into drone reliability, the lifespan and the usability of emergency drones in adverse weather conditions. Future work also involves training operators to fly and use the drone. This can be done in a staged approach using virtual drone simulators in digital environments leading to the supervised flying of drones in safe areas. Once trained the additional workload of the operators could be minimal. With 78 drone stations and 1400 OHCA events in Northern Ireland per annum, we can project that there would be less than 2 drone flights per month per drone station. If there are two trained operators, then this results in less than 1 drone flight per month per station.

Future work should also involve training and testing the ability of untrained bystanders to deliver successful defibrillation with accompanying human instructions with using public accessible AEDs.
